# Blood donation and health status based on SF-36: The mediating effect of cognition in blood donation

**DOI:** 10.1371/journal.pone.0223657

**Published:** 2019-10-22

**Authors:** Lerong Wang, Huimei Shi, Yanbo Zhu, Yanni Li, Xiaohan Yu, Muran Shi, Hui Yan, Tong Li, Jia Lu, Yanfeng Suo, Kun Zheng, Ooh Chye Tan

**Affiliations:** 1 School of Management, Beijing University of Chinese Medicine, Beijing, China; 2 School of Preclinical Medicine, Beijing University of Chinese Medicine, Beijing, China; University of Technology Sydney, AUSTRALIA

## Abstract

**Objective:**

The relationship among blood donation, cognition in blood donation and health condition of blood donors remains unclear. Based on our hypothesis, this study aimed to explore the mediating effect of cognition in blood donation on the relationship between blood donation and blood donors’ health status.

**Methods:**

A total of 837 participants who had prior experience in donating whole blood were recruited into a cross-sectional survey. The Medical Outcomes Study 36-Item Short-Form Health Survey (SF-36) and the Questionnaire on Cognition in Non-remunerated Blood Donation were used to evaluate the health status and the level of cognition in blood donation, respectively. Blood donation referred to the cumulative times of blood donation. The mediating effect of cognition in blood donation was analyzed by applying a path model.

**Results:**

The results revealed that blood donation was positively related to the physical component summary (PCS) and mental component summary (MCS) of SF-36, and cognition in blood donation was shown to have a partial mediating effect on the relationship between blood donation and both PCS and MCS. The effect size of cognition in blood donation was 24.63% in PCS and 26.72% in MCS.

**Conclusions:**

Blood donation is positively correlated with SF-36 outcomes (PCS and MCS) of blood donors, and cognition in blood donation plays a partial mediating effect in the relationship between blood donation and PCS and MCS.

## Introduction

Clinical blood transfusion is an indispensable method for disease treatment and saving the lives of patients suffering from blood loss; thus, the sufficient supply of clinical blood must be maintained [1, 2]. With the substantial increase in medical and health services, the demand for blood for clinical use has been on the rise. However, the recruitment of non-remunerated blood donors faces many issues at the present stage [3, 4]. In 2013, the rate of blood donation among residents in mainland China was 9.4 per thousand, which is lower than the minimum rate of blood donation of 10 per thousand recommended by World Health Organization (WHO). The composition of blood donors is considered too homogeneous, and the ability to retain repeating donors is weak. It is thus difficult for the quantity of donated blood to meet the growing demand for blood transfusions in China. Despite the progress made in raising public awareness of non-remunerated blood donation, many Chinese citizens still possess negative attitudes towards blood donation.

Previous studies have shown that appropriate and long-term blood donations are positively related to the physical and mental health of blood donors. Regular blood donations associated with pronounced decreases of blood pressure [5], lower values of some lipid profiles and higher Gamma Glutamic Transferase activity [6], and lower risk of cardiovascular disease [7]. In addition, regular donations could decrease the iron content in the human body, to a certain extent [8]. In the field of psychological research, Zhai [9] used the Symptom Checklist 90 (SCL-90) psychological scale to evaluate the mental status of non-remunerated blood donors and found that their mental health conditions are better and the levels of their anxiety, depression and fear are lower than those in the general population.

Cognition is defined as the mental action or process of acquiring knowledge and understanding through thought, experience, and the senses. The term is usually used to explain attitudes, the formation of knowledge, judgment and evaluation [10, 11]. Therefore, cognition in blood donation refers to one’s knowledge, attitude and judgment regarding blood donation. Some studies have shown that the act of blood donation is closely related to cognition in blood donation, which is increased in repeat blood donors [12]. In addition, an increased frequency in blood donation helps people grow accustomed to the environment and processes of the procedure, which alleviates their nervousness and uneasiness [13]. Using meta-cognitive psychological intervention, Wu [14] improved the cognitive level of blood donors and significantly reduced the rate of adverse reactions.

Therefore, the health condition of blood donors might be influenced by not only blood donation but also cognition in blood donation, which can be improved with the frequency of blood donations. Many researches have investigated the direct association between any two of blood donation, cognition in blood donation and blood donors’ health condition. Nevertheless, there might be an indirect association among them, which is rarely studied. Moderating effect and mediating effect are universal approaches used to investigate the direct and indirect effect of a third factor in the field of health [15]. Our previous studies showed that cognition in blood donation had moderating effect and mediating effect on the association between blood donation and blood donors’ health-related quality of life (HRQOL) [16, 17]. However, the studying sample of them included non-blood donors and blood donors with whole blood donation, blood component donation, and both. Consequently, the complexity of its research objects might result in miscalculation and puzzling results.

This study was conducted to explore the complex relationship between blood donation, cognition in blood donation and health condition of blood donors with whole blood donation only. We proposed a hypothesis that cognition in blood donation might act as a mediator and have a mediating effect between blood donation and blood donors’ health status. In other words, the relationship between blood donation and health status might be partially achieved through cognition in blood donation. A cross-sectional survey with whole blood donors was conducted to validate our hypothesis.

## Materials and methods

### Respondents

The cross-sectional study was based on field surveys and online surveys, which were carried out from May to September 2016. The field investigations were launched at the Beijing Red Cross blood center, which is one of the largest blood centers in China. This center was founded in 1957 with complete categories, advanced equipment, and a collection and supply of blood [18]. The respondents comprised of blood donors (those who donated whole blood and/or blood components) and non-donors. The selected participants were aged 18 to 60 years old (including the age of 18 and 60, according to the Blood Donation Law of the People’s Republic of China [19]). They were requested to sign informed consent forms and complete the questionnaires independently. Such informed consent forms were only needed for field surveys. Online respondents who agreed to participate in the survey proceeded with the survey after they acknowledged the purpose and recruitment criteria of this study but without signing explicit informed consents. Individuals with mental diseases, a history of chronic illnesses, or who failed to comprehend the contents of the questionnaires due to cognitive or sensory impairment were excluded.

### Ethical considerations

The study protocols were approved by the Clinical Research Ethics Review Committee of the Oriental Hospital of Beijing University of Chinese Medicine (No. JDF-IRB-2015030801). All participants were informed about the purpose of the research and all who were involved in the field survey signed the informed consent forms.

### Data collection

Data were collected by the utilization of structured questionnaires, which included the collection of demographic data, information on blood donation experiences, cognition in blood donation and health status. The social demography variables included gender, age, marital status (single, married or other), and education (below high school, high school or junior college, bachelor’s degree or above). These data were self-reported.

In accordance with the relevant contents of the Blood Donation Law of the People’s Republic of China, the minimum collection unit of whole blood is 200 ml (with a maximum collection unit of 400 ml). The interval between two blood donations must be at least six months [19]. The cumulative amount of blood donation and types of blood donation (whole blood donation only, blood component donation only, both whole blood donation and blood component donation) were self-reported by the respondents. Blood donation was represented by the cumulative times of blood donation (each 200 ml was recorded as one donation).

The cognitive status of blood donors was assessed by Cognition in Non-remunerated Blood Donation Questionnaire compiled by Zhu [20]. That questionnaire is a self-report instrument comprising 14 items in three dimensions, namely, positive cognition, negative cognition, and knowledge of blood donation. Participants were asked to rate their agreement or disagreement with each item on a 5-point Likert scale ranging from agree completely to disagree completely. Specific scoring methods were listed as follows: for the dimensions of positive cognition and knowledge of blood donation, the items were scored 1 (disagree completely) to 5 (agree completely). In contrast, the items scored 1 (agree completely) to 5 (disagree completely) for items of negative cognition. The original score of each dimension was the total score of its items. The standardized score of every dimension was determined as follows: (original score–the lowest possible score) / (the highest possible score–the lowest possible score) * 100; scores ranged from 0 to 100, with higher scores reflecting more positive cognition of blood donation. Cognition in blood donation contained the connotations of the three dimensions, was expressed by their average score. The Cronbach’s alpha coefficient (α = 0.841) revealed a high internal consistency [20].

HRQOL measures have been widely used to evaluate the health status for decades. To comprehensively evaluate blood donors’ physical status and well-being in this study, the health status was measured by generic HRQOL instrument Medical Outcomes Study 36-Item Short-Form Health Survey (SF-36, Chinese version 1.0)[21]. The SF-36 [22, 23] was made up of 36 questions that correspond to two domains and eight dimensions. The physical component summary (PCS) comprises four dimensions, namely, physical functioning (PF), role physical (RP), bodily pain (BP) and general health (GH). The mental component summary (MCS) includes vitality (VT), social functioning (SF), role emotional (RE) and mental health (MH). The SF-36 scores ranged from 0 to 100, with 100 representing the best state of health and 0 representing the worst. PCS and MCS were used to indicate scores of the physical domain and the mental domain, respectively, which could generalize the overall effect of physical and mental health status.

### Data analysis

Classified variables were described by using constituent ratios, while continuous variables were obtained by “mean ± SD (standard deviation)”. The Mann-Whitney U test and Kruskal-Wallis test (nonparametric test) were conducted to compare the differences between SF-36 scores and cognition in blood donation among blood donors with diverse characteristics. In addition, Cohen’s d and partial eta-squared (η_p_^2^) were used to measure the differences between groups of diverse characteristics to rule out the effect of sample size. Correlations between variables were studied using Spearman’s correlation. Statistical analyses were performed using SPSS version 25.0. Two-tailed P-values less than 0.05 were considered to be statistically significant.

We constructed a path model to test the hypothesis using Mplus version 8.0 and chose an appropriate method for parameter estimation, in accordance with the data type and normality of variables [24, 25]. A feasible path model should be based on related theories, and the fitting effect should achieve a reasonable standard. According to several major model fit indexes and in combination with the professional theory, nonsignificant parameters were sequentially removed, and significant parameters were added until the model fit achieved a standard level.

## Results

### Participant characteristics and SF-36 outcomes

A total of 1070 questionnaires were collected (887 from the field investigation and 183 through the online survey). After the deletion of 43 questionnaires with logical errors or missing data (drop-out rate: 4.02%), the sample at the end of investigation included a total of 1027 participants (857 from the field investigation and 170 through the online survey). Because the study was focused on blood donation (whole blood) and blood donors’ health conditions, 190 subjects who were non-donors or had ever contributed blood components at least one time were excluded from that batch of data. Ultimately, the sample size of the analysis was 837. As it is required that the sample size in a statistical analysis should be at least 5–10 times the number of variables, this sample met that requirement as there were 8 variables in this study [26, 27].

Among the respondents of this study, 546 (65.2%) were males and 291 (34.8%) were females (Table 1). The participants were aged from 18 to 60 years, with an average age of 30.8 years (SD = 9.3). A bachelor’s degree or above accounted for 55.1% of all respondents; single (49.8%) and married individuals (48.6%) each accounted for approximately half of all respondents. The average cumulative times of blood donations was 7.7±9.6.

**Table 1:** SF-36 outcomes and cognition in blood donation of blood donors with different characteristics.

Variable	N (%)	PCS				MCS				Cognition in blood donation			
		Mean±SD	T/F	P	Cohen’s d/ η_p_^2^	Mean± D	T/F	P	Cohen’s d/ η_p_^2^	Mean± D	T/F	P	Cohen’s d/ η_p_^2^
**Gender**													
Male	546(65.2)	91.6±8.8	3.25	<0.001	0.24	83.9±13.8	2.45	0.015	0.19	73.8±10.4	1.22	0.223	0.08
Female	291(34.8)	89.3±10.5				81.2±15.3				72.9±11.2			
**Age**													
≤30	490(58.5)	89.9±9.8	-3.13	<0.001	0.22	80.6±15.1	-6.00	<0.001	0.41	72.8±11.3	-2.48	0.013	0.17
>30	347(41.5)	92.0±9.0				86.3±12.6				74.6±9.8			
**Marital status**													
Single	417(49.8)	90.0±9.4	-2.27	0.024	0.16	80.1±15.2	-5.79	<0.001	0.40	72.5±11.1	-2.80	0.005	0.20
Married	407(48.6)	91.5±9.5				85.7±12.8				74.6±10.2			
**Education**													
Below high school	159(19.0)	92.5±9.2	8.33	<0.001	0.02	87.0±13.0	16.36	<0.001	0.04	72.6±9.0	0.84	0.432	0.00
High school or junior college	217(25.9)	92.1±7.7				85.2±12.5				73.5±9.5			
Bachelor’s degree or above	461(55.1)	89.6±10.2				80.5±15.1				73.8±11.7			

PCS: physical component summary; MCS: mental component summary; SD: standard deviation. Nonparametric Test and Cohen’s d were used to analyze the differences between groups of gender, age or marital status and their effect size, respectively. Kruskal-Wallis Test and η_p_^2^ were used to analyze the differences between groups of education and its effect size. Individuals of other marital status (13) were excluded in analysis.

The PCS and MCS scores of males were higher than those of females (PCS: 91.6±8.8 VS 89.3±10.5, P<0.001; MCS: 83.9±13.8 VS 81.2±15.3, P = 0.015; Table 1), but there were no significant differences in blood donation cognition between males and females (73.8±10.4 VS 72.9±11.2, P = 0.223, Table 1). For the other characteristics, the PCS, MCS and cognition scores of older participants (>30 years old) were higher than those of the younger participants (≤30 years old) (P<0.001, P<0.001, and P = 0.013 respectively, Table 1). The PCS, MCS and cognition scores of unmarried blood donors were lower than those of the married donors (P = 0.024, P<0.001, and P = 0.005 respectively, Table 1). The scores of PCS and MCS were significantly different among educational levels (both P<0.001, Table 1), while cognition in blood donation was not (P = 0.432, Table 1). From the point of effect size, sociodemographic variables had little effect on dependent variables (Cohen’s d: small effect≈0.2; medium effect≈0.5; big effect≈0.8 / / η_p_^2^: small effect<0.06)

### Correlation analysis

Significant positive correlations were found between cumulative times of blood donation and cognition in blood donation, as illustrated through the PCS and MCS scores (r = 0.22, P<0.01; r = 0.14, P<0.01; r = 0.19, P<0.01; Table 2). There was a significant positive correlation between scores of cognition and PCS (r = 0.17, P<0.01), and a positive correlation between scores of cognition and MCS (r = 0.19, P<0.01, Table 2).

**Table 2:** Correlation matrix of model variables.

	2	3	4
**1 Cumulative times of blood donation**	0.22**	0.14**	0.19**
**2 Cognition in blood donation**	-	0.17**	0.19**
**3 PCS**	-	-	0.58**
**4 MCS**	-	-	-

PCS: physical component summary; MCS: mental component summary.

** p<0.01 (both sides).

- indicates no correlation coefficient or repeated calculations.

### Mediating effects of cognition in blood donation

**Fig 1:**
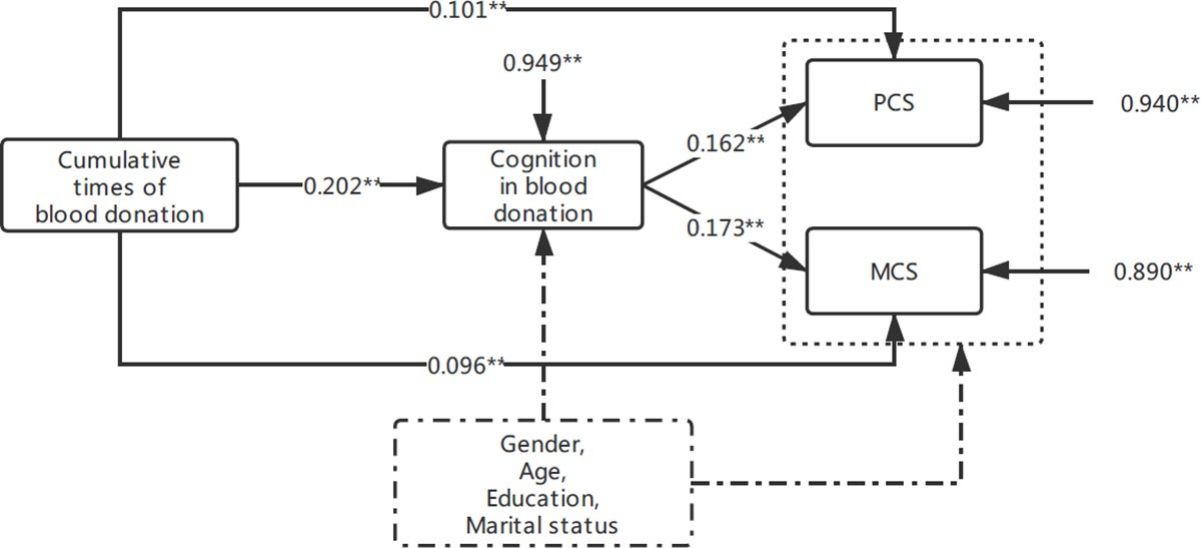
Path diagram for the mediating effect of cognition in blood donation. Values represent standardized path coefficients by a path analysis using Mplus 8.0 employing the S-B robust estimation method. Path coefficients are presented. P value for the path: ** p <0.01 (both sides).

We applied the S-B robust estimation [26, 27] due to the abnormal distribution of the cumulative times of blood donations, PCS, MCS and cognition in blood donation. In the initial path model, the cumulative times of blood donations, scores of cognition in blood donation, PCS and MCS were included to analyze the mediating effect of cognition in blood donation. The results demonstrated that the initial model was not ideal. Demographic characteristics such as gender, age, educational level and marital status were incorporated into the path model as controlled variables. The initial model was modified according to the test results of the model parameters. The fitting results are shown in Table 3. The comparative fit index (CFI) = 0.993 (>0.90), the Tucker-Lewis index (TLI) = 0.950 (>0.90), the root mean square error of approximation (RMSEA) = 0.043 (<0.08), the standardized root mean square residual (SRMR) = 0.013 (<0.08), and the weighted residual root mean residual (WRMR) = 0.494 (<1.00) were all in line with the ideal criterion. The final path was shown in Fig 1. The arrows in the model indicate the direction of the hypothetical association, and the values represent the strength of the path, namely, the standardized path coefficients. To reduce the influence of imbalance in sample size on the results, individuals with a marital status marked as “other” (13, 1.55%) were not included in the path analysis.

**Table 3:** The goodness of fit and judgment of mediating effects.

Model and standard	CFI	TLI	RMSEA	SRMR	WRMR
correction model	0.993	0.950	0.043	0.013	0.494
criterion for judging	>0.90	>0.90	<0.08	<0.08	<1.00

CFI: comparative fit index; TLI: Tucker-Lewis index; RMSEA: root mean square error of approximation; SRMR: standardized root mean square residual; WRMR: weighted residual root mean residual.

Cognition in blood donation had a partial mediating effect on PCS: the mediating effect amounted to 24.63% (Table 4). It can be interpreted as follows: when the cumulative times of blood donations increased by one unit, the score of PCS would increase by 0.134 units with 0.033 units caused by the indirect effect of cognition in blood donation. The cognition in blood donation also presented a partial mediating effect in MCS amounted to 26.72% (Table 4).

**Table 4:** Decomposition of effects of blood donation on PCS and MCS.

Model path	Direct effect	Indirect effect	Total effect	Mediating effect quantity (%)
Cumulative times of blood donation →cognition in blood donation →PCS	0.101**	0.033**	0.134**	24.63
Cumulative times of blood donation →cognition in blood donation →MCS	0.096**	0.035**	0.131**	26.72

PCS: physical component summary; MCS: mental component summary.

** p<0.01 (both sides).

## Discussion

This cross-sectional study with 837 whole blood donors explored the complex correlation between blood donation, cognition in blood donation and health status evaluated by SF-36 based on a mediating hypothesis. The main findings of this study were that the cumulative times of blood donation were positively related to SF-36 outcomes (both PCS and MCS) and that cognition in blood donation played a mediating effect on the relationship between blood donation and PCS and MCS. These findings thus confirmed our research hypothesis.

The cumulative times of blood donations were positively related to PCS and MCS scores, which meant that frequent and long-term non-remunerated blood donation was positively related to better health status, both in physical and psychological components. This finding coincided with our previous study that the HRQOL of blood donors gradually increased with donation times [16, 17, 28]. Moreover, a number of studies reported that frequent blood donation was positively related to blood donors’ health from different perspectives. For example, Meyers [29] conducted a retrospective cohort study and found that frequent blood donations were positively related to reduced risks of cardiovascular diseases. Zheng [30] attempted to assess the effects of frequency of blood donation on physiological and biochemical parameters such as iron reserve and vascular function. The results of that study revealed that donors who donated blood frequently had lower iron storage, lower oxidative stress and improved vascular function, in comparison with those donors with a lower frequency of blood donation. Similar results were also presented in Ositadinma’s study [31]. However, a recent study [32] in UK reported that over 2 years, more frequent donation could substantially help to collect more blood without having a major effect on donors’ HRQOL, physical activity, or cognitive function, but resulted in more donation-related symptoms, deferrals, and iron deficiency. Its inter-donation intervals for men were 12-week (standard), 10-week or 8-week, and for women were 16-week (standard), 14-week or 12-week. Besides, one collection unit is 470 ml of blood per session in UK which is quite different from that in China. More frequent donation and more amount of blood donation for one unit in UK than China might be one explanation for the difference of results in HRQOL between that study and our findings. Accordingly, it is important to explore the appropriate inter-donation interval and amount of blood donation for sufficient blood collection, which are not harmful to blood donors’ health at least or even helpful.

The results of mediating effect analysis indicated that cognition in blood donation played an intermediary role in the relationship between blood donation and SF-36 scores in both PCS and MCS. In other words, the cumulative times of blood donations had direct effect and indirect effect on blood donors’ health through cognition in blood donation. This outcome thus confirmed the research hypothesis. The mediating effect of cognition in blood donation in PCS was 24.63%, which illustrates that 24.63% of overall physical health benefits obtained through blood donation were caused by cognition in blood donation. At the same time, the mediating effect in MCS was 26.72%. A similar result of PCS (23.78%) was found while MCS was overestimated (32.43%) in our previous research [16] with a sample of subjects including both blood donors and non-blood donors. The HRQOL of blood donors was much better than non-blood donors [16, 28] and that of population norms [32–35] and difference of MCS scores was greater than that of PCS [28], then it might contribute to an overestimation of the mediating effect (especially in MCS) with participants including both blood donors and non-blood donors.

Previous studies [36, 37] also found that there was a close association between blood donation and cognition in blood donation and that the cognitive level of repeat blood donors was remarkably higher than that of first-time blood donors or non-donors. The majority of previous studies use the Knowledge-Attitude-Practice (KAP) model as their theoretical framework to explore the influence of cognition in blood donation on the act of blood donation [38, 39]. Nevertheless, blood donation itself could also contribute to the influencing of cognition in blood donation. First-time donation is normally carried out under the premise of preliminary knowledge of blood donation and relevant policies, while cognition merely remains at a theoretical level. In an attempt to explore the retention strategies for veteran blood donors, Ringwald [40] found that an individual’s comfortable experience and favorable feelings towards blood donation are important drivers for their repeat behaviors of blood donation. A comfortable experience during blood donation could be helpful in reducing the misconceptions about the safety of blood donation; meanwhile, the levels of cognition in blood donation and self-efficacy could also be improved. Though negative reactions occurred among some blood donors during or after blood donation [41], most of them have occurred among young blood donors or first-time blood donors. With the increase in the frequency of blood donation, the possibility of the occurrence of adverse reactions decreases gradually [42]. The cognition level of most of the repeat blood donors deepened and became more positive in conjunction with their comfortable experiences during blood donation. Ringwald’s study [40] also discovered that the number of repeat-blood-donor males was twice that of repeat-blood-donors females, and males generally believe that blood donation was beneficial in terms of health, thus generating a more positive cognition in blood donation.

### Limitations

There were some limitations in this study. First, data were collected through a self-reported structured form. Therefore, there might be a recall bias regarding the cumulative times of blood donations. Second, this study was a cross-sectional research study, which could not be used to infer causality. Furthermore, the quality and authenticity of the data obtained from the online survey might be lower than those collected via the field investigation. In addition, we did not investigate further as to whether the responding donors had ever been paid for donating blood. In terms of the interpretation of results, we could not be sure that the improvement in blood donors’ health was completely due to multiple blood donations. In summary, weaknesses were present in the study and further studies are needed to affirm the conclusions.

## Conclusions

In summary, this study analyzed the mediating effect of cognition in blood donation on the relationship between blood donation and health status measured by SF-36. We found that both the PCS and MCS of donors gradually increase with a rising frequency of blood donation and that cognition in blood donation plays a partial mediating effect between blood donation and SF-36 outcomes (PCS and MCS). The study enriches the theory that blood donation is beneficial to health.

## Supporting information

S1 File Additional tables. Participants’ responses to the Likert scales (Cognition in Non-remunerated Blood Donation Questionnaire and Medical Outcomes Study 36-Item Short-Form Health Survey) of individual statements.
(DOCX)

S2 File Initial data. (XLSX)

S3 File Editorial certificate (AJE). (PDF)
