# Supplementary material for: Blood donation and health status based on SF-36: The mediating effect of cognition in blood donation
Source: PLoS One. 2019 Oct 22;14(10):e0223657. doi: 10.1371/journal.pone.0223657 (PMC6804979; doi:10.1371/journal.pone.0223657)
Supplement: S1 File — Participants’ responses to the Likert scales (Cognition in Non-remunerated Blood Donation Questionnaire and Medical Outcomes Study 36-Item Short-Form Health Survey) of individual statements. (DOCX) [file pone.0223657.s001.docx]

**Cognition in Blood Donation Survey**

Cognition in Non-remunerated Blood Donation Questionnaire compiled by Zhu et al is a self-report instrument utilized to assess cognitive status of non-remunerated donors, comprising three dimensions: positive cognition, negative cognition, together with knowledge of blood donation, amounted to 14 items. Participants were asked to rate their agreement or disagreement with each item on a 5-point Likert scale ranging from 1 (agree completely) to 5 (disagree completely).

Table 1. Participants’ responses to Cognition in Blood Donation Survey in dimension of **positive cognition**

| Item | 1.Agree completely | |  | 2. Agree | |  | 3.Uncertain | |  | 4.Disagree | |  | 5. Disagree completely | |
| --- | --- | --- | --- | --- | --- | --- | --- | --- | --- | --- | --- | --- | --- | --- |
|  | N | % |  | N | % |  | N | % |  | N | % |  | N | % |
| q1.Blood donation can help us know our health status | 257 | 30.70 |  | 504 | 60.22 |  | 56 | 6.69 |  | 17 | 2.03 |  | 3 | 0.36 |
| q2.Blood donation can make it easier for the family to use blood | 180 | 21.50 |  | 558 | 66.67 |  | 79 | 9.44 |  | 17 | 2.03 |  | 3 | 0.36 |
| q3.Blood donation is safe | 226 | 27.00 |  | 581 | 69.41 |  | 25 | 2.99 |  | 4 | 0.48 |  | 1 | 0.12 |
| q4. Blood donation can save the lives of others | 289 | 34.53 |  | 524 | 62.60 |  | 19 | 2.27 |  | 3 | 0.36 |  | 2 | 0.24 |
| q5.Blood donors can earn the respect of others | 135 | 16.12 |  | 454 | 54.24 |  | 197 | 23.54 |  | 47 | 5.62 |  | 4 | 0.48 |
| q6. Blood donation is a meaningful thing | 267 | 31.90 |  | 550 | 65.71 |  | 17 | 2.03 |  | 3 | 0.36 |  | 0 | 0.00 |
| q7. I can donate blood anytime if necessary | 219 | 26.16 |  | 544 | 65.00 |  | 53 | 6.33 |  | 18 | 2.15 |  | 3 | 0.36 |

Note: The items were scored reversely [ 1 (disagree completely) to 5 (agree completely)].

Table 2. Participants’ responses to Cognition in Blood Donation Survey in dimension of **negative cognition**

| Item | 1.Agree completely | |  | 2. Agree | |  | 3.Uncertain | |  | 4.Disagree | |  | 5. Disagree completely | |
| --- | --- | --- | --- | --- | --- | --- | --- | --- | --- | --- | --- | --- | --- | --- |
|  | N | % |  | N | % |  | N | % |  | N | % |  | N | % |
| q8. Blood donation is harmful to health | 1 | 0.12 |  | 14 | 1.67 |  | 121 | 14.46 |  | 573 | 68.46 |  | 128 | 15.29 |
| q9. Blood donation may cause anemia | 2 | 0.24 |  | 32 | 3.82 |  | 182 | 21.75 |  | 519 | 62.00 |  | 102 | 12.19 |
| q10. Blood donation may spread diseases | 6 | 0.72 |  | 101 | 12.07 |  | 200 | 23.88 |  | 446 | 53.29 |  | 84 | 10.04 |
| q11. Blood donation may waste time | 2 | 0.24 |  | 39 | 4.66 |  | 60 | 7.17 |  | 647 | 77.30 |  | 89 | 10.63 |
| q12.Blood donation scares me | 5 | 0.60 |  | 44 | 5.26 |  | 53 | 6.33 |  | 622 | 74.31 |  | 113 | 13.50 |

Note: The items were scored forward [1 (agree completely) to 5 (disagree completely)].

Table 3. Participants’ responses to Cognition in Blood Donation Survey in dimension of **knowledge of blood donation**

| Item | 1.Agree completely | |  | 2. Agree | |  | 3.Uncertain | |  | 4.Disagree | |  | 5. Disagree completely | |
| --- | --- | --- | --- | --- | --- | --- | --- | --- | --- | --- | --- | --- | --- | --- |
|  | N | % |  | N | % |  | N | % |  | N | % |  | N | % |
| q13. I know what to pay attention to before donating blood | 124 | 14.81 |  | 495 | 59.14 |  | 161 | 19.24 |  | 55 | 6.57 |  | 2 | 0.24 |
| q14. I know what to pay attention to after donating blood | 126 | 15.05 |  | 494 | 59.02 |  | 160 | 19.12 |  | 56 | 6.69 |  | 1 | 0.12 |

Note: The items were scored reversely [ 1 (disagree completely) to 5 (agree completely)].

**Health-related Quality of Life**

HRQOL measures have been widely used to evaluate the health status for decades. To comprehensively evaluate blood donors’ physical status and well-being in this study, the health status was measured by generic HRQOL instrument Medical Outcomes Study 36-Item Short-Form Health Survey (SF-36, Chinese version 1.0). The SF-36 was made up of 36 questions that correspond to two domains and eight dimensions. The physical component summary (PCS) comprises four dimensions, namely, physical functioning (PF), role physical (RP), bodily pain (BP) and general health (GH). The mental component summary (MCS) includes vitality (VT), social functioning (SF), role emotional (RE) and mental health (MH). The SF-36 scores ranged from 0 to 100, with 100 representing the best state of health and 0 representing the worst. PCS and MCS were used to indicate scores of the physical domain and the mental domain, respectively, which could generalize the overall effect of physical and mental health status. Please refer to the contents of items and scoring methods of the version 1.0 in English.

**Physical component summary (PCS)**

Table 4. Participants’ responses to MOS 36-item short-form health survey in dimension of **physical functioning (PF)**

| Item | 1.Yes, limited a lot | |  | 2.Yes, limited a little | |  | 3.No, not limited at all | |
| --- | --- | --- | --- | --- | --- | --- | --- | --- |
|  | N | % |  | N | % |  | N | % |
| q3a.Vigorous activities, such as running, lifting heavy objects, participating in strenuous sports | 20 | 2.39 |  | 189 | 22.58 |  | 628 | 75.03 |
| q3b.Moderate activities, such as moving a table, pushing a vacuum cleaner, bowling, or playing golf | 0 | 0.00 |  | 23 | 2.75 |  | 814 | 97.25 |
| q3c. Lifting or carrying groceries | 0 | 0.00 |  | 12 | 1.43 |  | 825 | 98.57 |
| q3d. Climbing several flights of stairs | 0 | 0.00 |  | 34 | 4.06 |  | 803 | 95.94 |
| q3e. Climbing one flight of stairs | 0 | 0.00 |  | 4 | 0.48 |  | 833 | 99.52 |
| q3f. Bending, kneeling, or stooping | 1 | 0.12 |  | 27 | 3.23 |  | 809 | 96.65 |
| q3g. Walking more than a mile | 1 | 0.12 |  | 17 | 2.03 |  | 819 | 97.85 |
| q3h. Walking several blocks | 0 | 0.00 |  | 6 | 0.72 |  | 831 | 99.28 |
| q3i. Walking one block | 0 | 0.00 |  | 1 | 0.12 |  | 836 | 99.88 |
| q3j. Bathing or dressing yourself | 0 | 0.00 |  | 3 | 0.36 |  | 834 | 99.64 |

Note: The above items are about activities you might do during a typical day. Does your health now limit you in these activities? If so, how much? The items were scored forward.

Table 5. Participants’ responses to MOS 36-item short-form health survey in dimension of **role physical (RP)**

| Item | 1.Yes | |  | 2.No | |
| --- | --- | --- | --- | --- | --- |
|  | N | % |  | N | % |
| q4a. Cut down the amount of time you spent on work or other activities | 43 | 5.14 |  | 794 | 94.86 |
| q4b. Accomplished less than you would like | 57 | 6.81 |  | 780 | 93.19 |
| q4c. Were limited in the kind of work or other activities | 42 | 5.02 |  | 795 | 94.98 |
| q4d. Had difficulty performing the work or other activities (for example, it took extra effort) | 40 | 4.78 |  | 797 | 95.22 |

Note: These questions are about (During the past 4 weeks, have you had any of the following problems with your work or other regular daily

activities as a result of your physical health? ). The items were scored forward.

Table 6. Participants’ responses to MOS 36-item short-form health survey in dimension of **bodily pain (BP)**

| Item | 1. None | |  | 2. Very mild | |  | 3. Mild | |  | 4. Moderate | |  | 5. Severe | |  | 6. Very severe | |
| --- | --- | --- | --- | --- | --- | --- | --- | --- | --- | --- | --- | --- | --- | --- | --- | --- | --- |
|  | N | % |  | N | % |  | N | % |  | N | % |  | N | % |  | N | % |
| q7. How much bodily pain have you had during the past 4 weeks? | 548 | 65.47 |  | 155 | 18.52 |  | 108 | 12.90 |  | 20 | 2.39 |  | 5 | 0.60 |  | 1 | 0.12 |
|  |  |  |  |  |  |  |  |  |  |  |  |  |  |  |  |  |  |
|  | 1. Not at all | |  | 2. A little bit | |  | 3. Moderately | |  | 4. Quite a bit | |  | 5. Extremely | |  |  |  |
|  | N | % |  | N | % |  | N | % |  | N | % |  | N | % |  |  |  |
| q8. During the past 4 weeks, how much did pain interfere with your normal work (including both work outside the home and housework)? | 715 | 85.42 |  | 110 | 13.14 |  | 8 | 0.96 |  | 4 | 0.48 |  | 0 | 0.00 |  |  |  |

Note: The items (q7 and q8) were scored reversely.

Table 7. Participants’ responses to MOS 36-item short-form health survey in dimension of **general health (GH)**

| Item | 1. Excellent | |  | 2. Very good | |  | 3. Good | |  | 4. Fair | |  | 5. Poor | |
| --- | --- | --- | --- | --- | --- | --- | --- | --- | --- | --- | --- | --- | --- | --- |
|  | N | % |  | N | % |  | N | % |  | N | % |  | N | % |
| q1. How much bodily pain have you had during the past 4 weeks? | 213 | 25.45 |  | 364 | 43.49 |  | 172 | 20.55 |  | 87 | 10.39 |  | 1 | 0.12 |
|  |  |  |  |  |  |  |  |  |  |  |  |  |  |  |
|  | 1.Definitely true | |  | 2.Mostly true | |  | 3. Don't know | |  | 4. Mostly false | |  | 5.Definitely false | |
|  | N | % |  | N | % |  | N | % |  | N | % |  | N | % |
| q10a.I seem to get sick a little easier than other people. | 1 | 0.12 |  | 28 | 3.35 |  | 117 | 13.98 |  | 157 | 18.75 |  | 534 | 63.80 |
| q10b. I am as healthy as anybody I know. | 315 | 37.64 |  | 352 | 42.06 |  | 108 | 12.90 |  | 48 | 5.73 |  | 14 | 1.67 |
| q10c.I expect my health to get worse. | 15 | 1.79 |  | 67 | 8.00 |  | 166 | 19.83 |  | 143 | 17.08 |  | 446 | 53.30 |
| q10d.My health is excellent. | 412 | 49.22 |  | 316 | 37.75 |  | 88 | 10.51 |  | 16 | 1.92 |  | 5 | 0.60 |

Note: These questions are about how TRUE or FALSE is each of the following statements for you. The items (q1, q10b and q10d) were scored reversely. The items (q10a and q10c) were scored forward.

**Mental component summary (MCS)**

Table 8. Participants’ responses to MOS 36-item short-form health survey in dimension of **vitality (VT)**

| Item | 1. All of the time | |  | 2. Most of the time | |  | 3. A good bit of the time | |  | 4. Some of the time | |  | 5. A little of the time | |  | 6. None of the time | |
| --- | --- | --- | --- | --- | --- | --- | --- | --- | --- | --- | --- | --- | --- | --- | --- | --- | --- |
|  | N | % |  | N | % |  | N | % |  | N | % |  | N | % |  | N | % |
| q9a. Did you feel full of pep? | 174 | 20.79 |  | 443 | 51.73 |  | 115 | 13.74 |  | 63 | 7.53 |  | 11 | 1.31 |  | 31 | 3.70 |
| q9e. Did you have a lot of energy? | 176 | 21.03 |  | 392 | 46.83 |  | 165 | 19.71 |  | 66 | 7.89 |  | 26 | 3.11 |  | 12 | 1.43 |
| q9g. Did you feel worn out? | 4 | 0.48 |  | 15 | 1.79 |  | 26 | 3.11 |  | 85 | 10.16 |  | 314 | 37.51 |  | 393 | 46.95 |
| q9i. Did you feel tired? | 0 | 0.00 |  | 29 | 3.46 |  | 40 | 4.78 |  | 125 | 14.93 |  | 351 | 41.94 |  | 292 | 34.89 |

Note: These questions are about how you feel and how things have been with you during the past 4 weeks. For each question, please give the one answer that comes closest to the way you have been feeling. How much of the time during the past 4 weeks...The items (q9a and q9e) were scored reversely. The items (q9g and q9i) were scored forward.

Table 9. Participants’ responses to MOS 36-item short-form health survey in dimension of **social functioning (SF)**

| Item | 1. Not at all | |  | 2. Slightly | |  | 3. Moderately | |  | 4. Quite a bit | |  | 5. Extremely | |  |  | |
| --- | --- | --- | --- | --- | --- | --- | --- | --- | --- | --- | --- | --- | --- | --- | --- | --- | --- |
|  | N | % |  | N | % |  | N | % |  | N | % |  | N | % |  |  |  |
| q6. During the past 4 weeks, to what extent has your physical health or emotional problems interfered with your normal social activities with family, friends, neighbors, or groups? | 584 | 69.78 |  | 201 | 24.01 |  | 38 | 4.54 |  | 12 | 1.43 |  | 2 | 0.24 |  |  |  |
|  |  |  |  |  |  |  |  |  |  |  |  |  |  |  |  |  |  |
|  | 1. All of the time | |  | 2. Most of the time | |  | 3. A good bit of the time | |  | 4. Some of the time | |  | 5. A little of the time | |  | 6.None of the time | |
|  | N | % |  | N | % |  | N | % |  | N | % |  | N | % |  | N | % |
| q9j. During the past 4 weeks, how much of the time has your physical health or emotional problems interfered with your social activities (like visiting with friends, relatives, etc.)? | 9 | 1.07 |  | 3 | 0.36 |  | 14 | 1.79 |  | 24 | 2.86 |  | 87 | 10.50 |  | 700 | 83.42 |

Note: The item (q6) was scored reversely. The item (q9i) was scored forward.

Table 10. Participants’ responses to MOS 36-item short-form health survey in dimension of **role emotional (RE)**

| Item | 1.Yes | |  | 2.No | |
| --- | --- | --- | --- | --- | --- |
|  | N | % |  | N | % |
| q5a. Cut down the amount of time you spent on work or other activities. | 146 | 17.44 |  | 691 | 82.56 |
| q5b Accomplished less than you would like. | 151 | 18.04 |  | 686 | 81.96 |
| q5c. Didn't do work or other activities as carefully as usual. | 160 | 19.12 |  | 677 | 80.88 |

Note: These questions are about during the past 4 weeks, have you had any of the following problems with your work or other regular daily

activities as a result of any emotional problems (such as feeling depressed or anxious)? The items were scored forward.

Table 11. Participants’ responses to MOS 36-item short-form health survey in dimension of **mental health (MH)**

| Item | 1. All of the time | |  | 2. Most of the time | |  | 3. A good bit of the time | |  | 4. Some of the time | |  | 5. A little of the time | |  | 6.None of the time | |
| --- | --- | --- | --- | --- | --- | --- | --- | --- | --- | --- | --- | --- | --- | --- | --- | --- | --- |
|  | N | % |  | N | % |  | N | % |  | N | % |  | N | % |  | N | % |
| q9b. Have you been a very nervous person? | 2 | 0.24 |  | 33 | 3.94 |  | 58 | 6.93 |  | 125 | 14.94 |  | 257 | 30.70 |  | 362 | 43.25 |
| q9c. Have you felt so down in the dumps that nothing could cheer you up? | 2 | 0.24 |  | 10 | 1.19 |  | 14 | 1.67 |  | 78 | 9.32 |  | 242 | 28.91 |  | 491 | 58.67 |
| q9d.Have you felt calm and peaceful? | 143 | 17.08 |  | 380 | 45.40 |  | 122 | 14.58 |  | 100 | 11.95 |  | 49 | 5.85 |  | 43 | 5.14 |
| q9f. Have you felt downhearted and blue? | 5 | 0.60 |  | 14 | 1.67 |  | 26 | 3.11 |  | 88 | 10.51 |  | 373 | 44.56 |  | 331 | 39.55 |
| q9h. Have you been a happy person? | 233 | 27.83 |  | 421 | 50.30 |  | 102 | 12.19 |  | 40 | 4.78 |  | 27 | 3.23 |  | 14 | 1.67 |

Note: These questions are about how you feel and how things have been with you during the past 4 weeks. For each question, please give the one answer that comes closest to the way you have been feeling. How much of the time during the past 4 weeks....The items (q9d and q9h) were scored reversely. The items (q9b, q9c and q9f) were scored forward.
